# Capacity of *Nerium oleander* to Phytoremediate Sb-Contaminated Soils Assisted by Organic Acids and Oxygen Nanobubbles

**DOI:** 10.3390/plants12010091

**Published:** 2022-12-24

**Authors:** Petroula Seridou, Sofia Monogyiou, Evdokia Syranidou, Nicolas Kalogerakis

**Affiliations:** 1 School of Chemical and Environmental Engineering, Technical University of Crete, 73100 Chania, Greece; 2 Institute of Geoenergy, Foundation for Research and Technology-Hellas (FORTH), 73100 Chania, Greece

**Keywords:** *Nerium oleander*, antimony, phytoremediation, nanobubbles, organic acids

## Abstract

Antimony (Sb) is considered to be a toxic metalloid of increasing prevalence in the environment. Although several phytoremediation studies have been conducted, research regarding the mechanisms of Sb accumulation and translocation within plants remains limited. In this study, soil from a shooting range was collected and spiked with an initial Sb(III) concentration of 50 mg/kg. A pot experiment was conducted to investigate whether *Nerium oleander* could accumulate Sb in the root and further translocate it to the aboveground tissue. Biostimulation of the soil was performed by the addition of organic acids (OAs), consisting of citric, ascorbic, and oxalic acid at low (7 mmol/kg) or high (70 mmol/kg) concentrations. The impact of irrigation with water supplemented with oxygen nanobubbles (O_2_NBs) was also investigated. The results demonstrate that there was a loss in plant growth in all treatments and the presence of OAs and O_2_NBs assisted the plant to maintain the water content at the level close to the control. The plant was not affected with regards to chlorophyll content in all treatments, while the antioxidant enzyme activity of guaiacol peroxidase (GPOD) in the roots was found to be significantly higher in the presence of Sb. Results revealed that Sb accumulation was greater in the treatment with the highest OAs concentration, with a bioconcentration factor greater than 1.0. The translocation of Sb for every treatment was very low, confirming that *N. oleander* plant cannot transfer Sb from the root to the shoots. A higher amount of Sb was accumulated in the plants that were irrigated with the O_2_NBs, although the translocation of Sb was not increased. The present study provides evidence for the phytoremediation capacity of *N. oleander* to bioaccumulate Sb when assisted by biostimulation with OAs.

## 1. Introduction

Antimony (Sb) is a metalloid that belongs to Group 15 of the periodic table. It exists in four major oxidation states (−III, 0, III, and V); antimonite (state of +3, represented as Sb(III)) and antimonate (state of +5, represented as Sb(V)) are the most predominant species in the environment [1]. Sb is recognized as a priority pollutant, which can cause acute environmental issues since it is released into soils and aquatic environments by natural processes and mainly by human activities such as mining, coal combustion, and shooting [2]. The pollution of this metalloid is rapidly emerging worldwide due to its extensive use, especially in China, which is the leading producer of Sb [3]. Apart from the environmental risk, Sb is considered hazardous to human health, as it is a suspected carcinogen due to its toxicity [4]. Specifically, trivalent compounds of antimony have been found to be more toxic (10 times) than the pentavalent ones [5]. Sb removal from environmental water bodies has received a great deal of attention in the last decades; therefore, technologies such as coagulation, adsorption, and the electrochemical method have been widely tested and found to be effective [6,7,8]. However, the main disadvantages of these methods are the use of chemicals, the high energy consumption, and the risk of secondary pollution; hence, the efficient removal of the antimony compounds from the environment remains a challenge [9].

In soil, Sb is mostly encountered in the forms of Sb(III) and Sb(V) and the latter shows higher water solubility. Sb has been reported to exceed the value of 5000 mg/kg when background concentration in the natural environment is only 0.2 mg/kg, and the maximum permissible concentration according to the World Health Organization (WHO) is set at 36 mg/kg [3,10,11]. The predominant species are Sb(V) under oxic conditions and Sb(III) under reducing conditions [12]. In active Sb mining areas, a high Sb fraction is bioavailable, comprised primarily of Sb(V) [13]. At shooting ranges, the leading form of Sb is noted as Sb(V) due to the relatively fast oxidation of Sb(III) [14]. Previous studies suggest that the trivalent neutral complex Sb(OH)_3_ is sorbed to Fe (hydro)oxides over a wide range of pH, and hence Sb(III) is considered immobile in neutral soils [15].

A technology that can be employed to manage Sb pollution at contaminated sites is phytoremediation using plant species that can accumulate antimony at a high level [16]. Previous studies have shown that plants display different abilities to uptake the various forms of Sb speciation. In general, Sb(III) is easily oxidized into Sb(V) in the soil environment, while plants can accumulate both Sb(III) and Sb(V). Specifically, several studies have indicated that plants display higher affinity to sorb more Sb(III) than Sb(V) [17,18,19]. On the contrary, Shtangeeva et al. [20] recorded that rye absorbed a higher amount of Sb(V) than Sb(III). *Pteris cretica var. nervosa* has been investigated for Sb phytoremediation and was found to be a Sb hyperaccumulator with no high translocation from root to shoots. When the test plant was exposed to 500 mg/kg of Sb, 672.8 mg Sb/kg were accumulated in the plant while when the initial concentration was 1000 mg/kg, the plant uptake was estimated 2054.8 mg Sb/kg [21]. Seedlings of *S. bicolor* in Sb-contaminated soil were treated with different levels of TiO_2_ nanoparticles and the results showed that the bioconcentration factor was above one for each treatment, indicating that this plant also has phytoremediation potential [22]. To date, the mechanism of Sb accumulation in plants is not well understood and there is need for in-depth studies.

*Nerium oleander* is an evergreen shrub native to the Mediterranean region, which is grown as an ornamental plant of high aesthetic value. Moreover, this plant is salt tolerant and resistant to drought. Generally, it is able to tolerate high concentrations of heavy metals (HMs) in soil [23]. It has been shown to have the capability to accumulate HMs, thus making it a promising candidate for phytoremediation applications. In a recent study, Ibrahim and El Afandi [24] concluded that Cd and Zn were concentrated in the aerial parts of the *N. oleander* plant, while Pb was accumulated in the root. *N. oleander* has shown a good capacity to bioaccumulate the following metals: Pb, Cr, Cu, Li, Ni, and Zn [25].

Proper amendments can be applied to achieve optimal growth of plants and soil amelioration. One option is the addition of low molecular weight organic acids (OAs), which are typical root exudates for plants. The bioavailability of HMs in soils can be enhanced, since organic acids can solubilize metal oxides and assist the plant to uptake the contaminants from soil [26]. Due to the limited secretion of OAs by plant roots, adding exogenous OAs is an effective method to improve phytoremediation, since they are efficient chelating agents for the cleaning up of toxic HMs from soils [27,28]. In addition, oxygenation can increase the oxygen content and hence, the aerobic respiration of crop roots is improved, increasing enzyme activity in the soil. The aeration efficiency can be increased by the oxygen delivery via small sized gaseous bubbles [29,30]. Nanobubbles (NBs) are tiny spherical gaseous bodies sized at <1 μm in diameter, which exhibit unique properties, including long lifetime in aqueous solutions and high specific area, thanks to their small size. Nanobubble technology has attracted significant scientific interest in disinfection [31], flotation [32] and organic pollutant removal [33]. There are also several studies focusing on nanobubbles application in agriculture. Irrigation water containing NBs promoted higher germination rates in seeds [34,35,36,37] and had also a beneficial effect on plant growth [30,38].

As far as we know, no previous research has investigated the phytoremediation potential of *Nerium oleander* for antimony-contaminated soils. In this work, the ability of *N. oleander* to uptake, translocate, and tolerate Sb was examined in five treatments. The presence of 50 ppm Sb at various conditions in the soils was investigated (treatments B, C, D, and E) and compared to soil with a low background concentration of Sb (treatment A). In addition, the effect of supplementation with citric acid (CA), oxalic acid (OA), and ascorbic acid (AA) on Sb mobilization and total Sb accumulation was investigated (treatments C, D, and E). Finally, it was examined whether irrigation with oxygen nanobubbles (O_2_NBs) may enhance the phytoextraction of Sb by the plant (treatment D).

## 2. Results

The soil pH was measured in each treatment before and after the end of the experimental period and the values are listed below (Table 1). The initial pH of the soil was found to be 7.42. In the control treatment, the pH dropped to 7.17, while in the treatment with soil spiked with antimony, the pH was slightly increased to 7.51. The addition of OAs led to acidification of the soil. In particular, the addition of a low concentration of OAs reduced the soil pH to 6.61, while the highest concentration led to a pH of 6.04. Remarkably, after the end of the experiment, the pH of the soil was increased approximately to 7.65 and 7.23 in the low and high OA concentration treatments, respectively.

**Figure 1:**
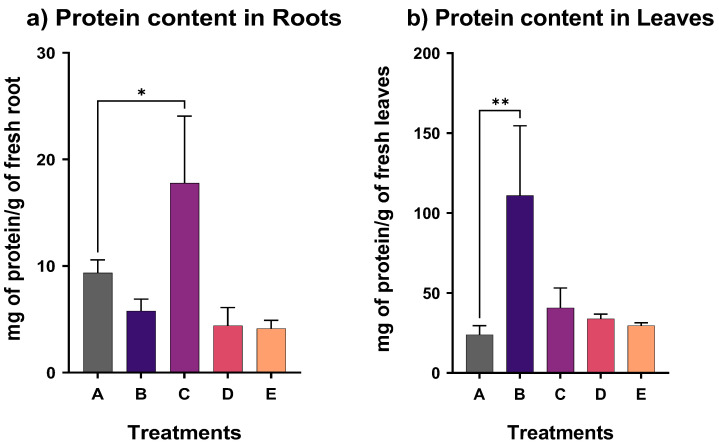
Protein content (mg protein/g FW) in (**a**) root and (**b**) leaves for all treatments (star indicates the level of significance: * for *p* < 0.05, ** for *p* < 0.01).

The protein content was examined in the root and leaves of *N. oleander* for each treatment. As shown in Figure 1, a significant difference was found between the root of treatment C, exposed to the antimony and the low concentration of OAs, and the control treatment. In particular, 9.4 mg protein per g fresh root was found in the control and 17.7 mg protein per g fresh root in treatment C. The protein concentration of leaves is considerably higher than in the root. The protein content of leaves was statistically different in treatment B from the control treatment. Notably, the protein content was found to be 23.8 mg/g of fresh leaves in the control and 110.9 mg/g in treatment B.

**Figure 2:**
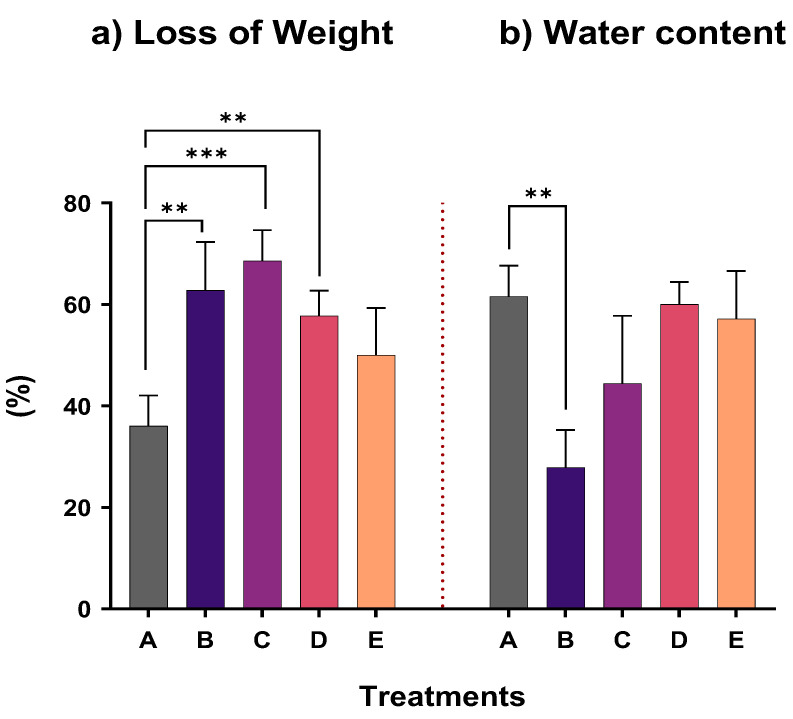
Loss of weight and water content at the end of the experiment for all treatments (star indicates the level of significance: ** for *p* < 0.01, *** for *p* < 0.001).

Physiological changes in plants were also evaluated by measuring the loss of fresh weight of roots and leaves and the water content (Figure 2). By examining the weight, in all treatments, a loss was observed, with the lowest percentage observed in the control treatment (A). Analysis among the treatments showed a significant decrease of weight in treatments B, C, and D. In treatment E, weight loss was also increased compared to the control, however no significant difference was observed. The plant water content was found to be adversely affected by the presence of antimony in treatment B compared to the control, since a statistical difference was observed. The plants in the other treatments exposed to Sb were not found to be significantly influenced. In treatments D and E, the water content was greatly close to the percentage of the control, while in treatment C, the water content was decreased, but not significantly.

**Figure 3:**
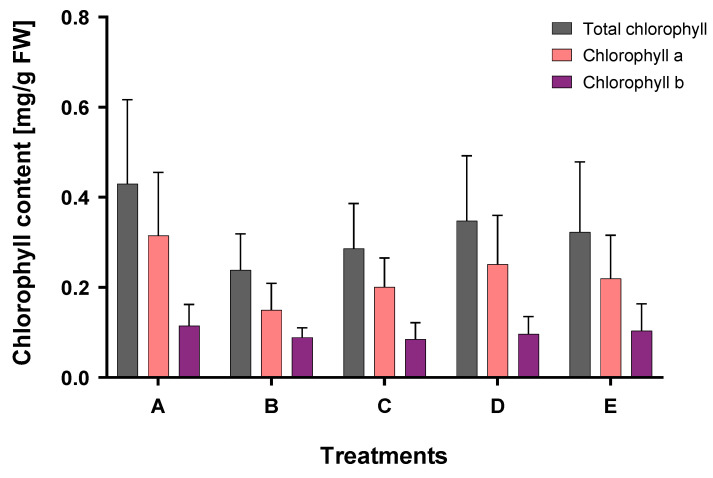
Chlorophyll (a, b, total) content in plant tissues (leaves) for all treatments at the end of the experiment.

Regarding total chlorophyll, chlorophyll a, and chlorophyll b, statistically significant differences compared to the control were not found for all treatments. As shown in Figure 3, the chlorophyll content (mg/g FW) was the highest in the control, while the lowest was found in treatment B, where no organic acids were added to the soil. In the case of the combination of OAs and O_2_NBs (treatment D), the chlorophyll content was found to be the highest among all treatments, excluding the control, although this difference is not statistically significant. Overall, the chlorophyll content in all treatments is not statistically different.

**Figure 4:**
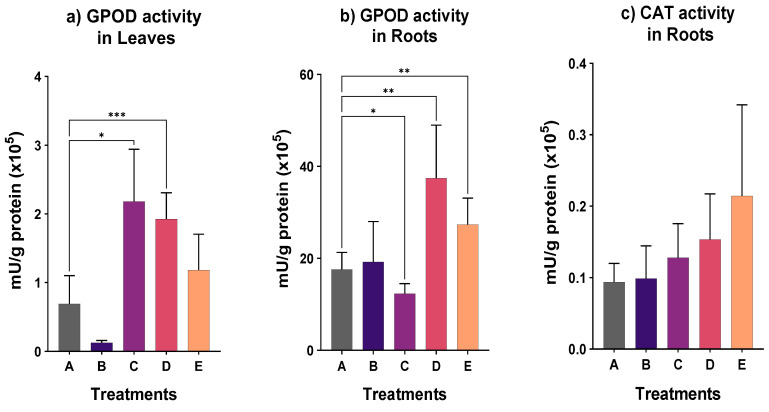
Specific guaiacol peroxidase in (**a**) leaves, (**b**) roots and (**c**) catalase activity in roots for all treatments (star indicates the level of significance: * for *p* < 0.05, ** for *p* < 0.01, *** *p* < 0.001).

The enzymes activity involved in antioxidant defense were determined in the root and leaves (Figure 4). Specifically, the catalase (CAT) activity in the root was not significantly affected by exposure to antimony, since no statistical difference was observed. The guaiacol peroxidase (GPOD) in the roots was significantly higher in treatments D and E compared to the control. The enzyme production in leaves from treatments C and D was significantly elevated compared to the activity of the enzyme in leaves of non-spiked soil. The level of GPOD activity was higher in roots than in leaves in all treatments. Only in treatment D, a significant increase in GPOD activity was recorded both in the root and the leaves.

**Figure 5:**
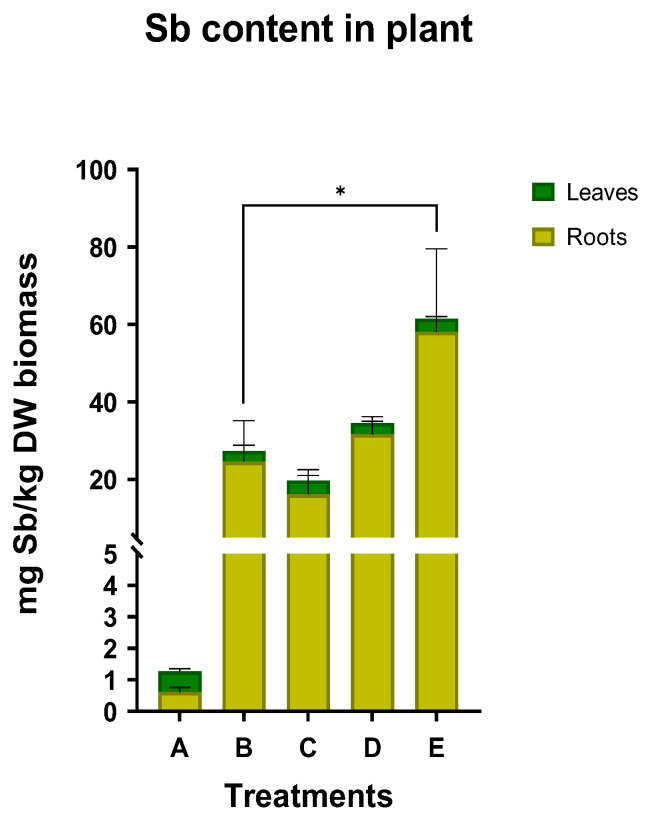
Sb accumulation in roots and leaves for all treatments (star indicates the level of significance: * for *p* < 0.05).

In Figure 5, the Sb content accumulated in roots and leaves is shown. In Sb-contaminated soils, the highest content was detected in treatment E, with 58.10 mg/kg DW biomass in the roots and 3.38 mg/kg DW biomass in the leaves. On the contrary, the lowest Sb concentration was found in treatment C, where again, the majority of the Sb remained in the roots. In the control with the background concentration of Sb, the accumulation was found to be 0.61 mg/kg DW biomass in the roots and 0.66 mg/kg DW biomass in the leaves. The estimated translocation factor was close to unity, much higher compared to all other treatments where Sb is present at a much higher concentration in the soil.

In treatment E, the bioconcentration factor was found to be >1, indicating that Sb can be transferred from the soil to the roots of *N. oleander* (Table 2). In all other treatments including the control, the bioconcentrations factors were found to be less than 1, indicating a low mobilization of Sb from the soil to the plant. Regarding the translocation factor, the values in all treatments have been found to be significantly lower than one, suggesting that Sb could not be readily transferred from the roots to the above-ground tissues. The TF in treatment C was significantly higher compared to all other treatments (0.23), which were significantly lower (<0.1).

**Figure 6:**
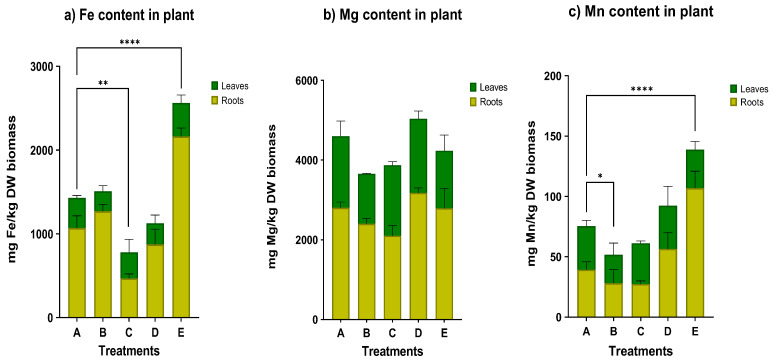
Concentration of (**a**) Fe, (**b**) Mg and (**c**) Mn in plant tissues for all treatments (star indicates the level of significance: * for *p* < 0.05, ** for *p* < 0.01, **** for *p* < 0.0001).

Besides the Sb concentration in plant tissues, Fe, Mg, and Mn uptake by *N. oleander* was also measured (Figure 6), since these elements are essential for plant growth. Regarding the Mg content, there is a small decrease in treatments B, C, and E, however these reductions are not statistically significant. On the other hand, Fe and Mn uptake by *N. oleander* was significantly promoted by the addition of OAs at a high concentration (70 mmol/kg). In treatment C, the accumulation of Fe was found to be statistically lower compared to the control. Overall, the concentration of Fe, Mg and Mn is much higher in roots, indicating a limited translocation from roots to shoots, apart from Mn content in treatment C.

**Figure 7:**
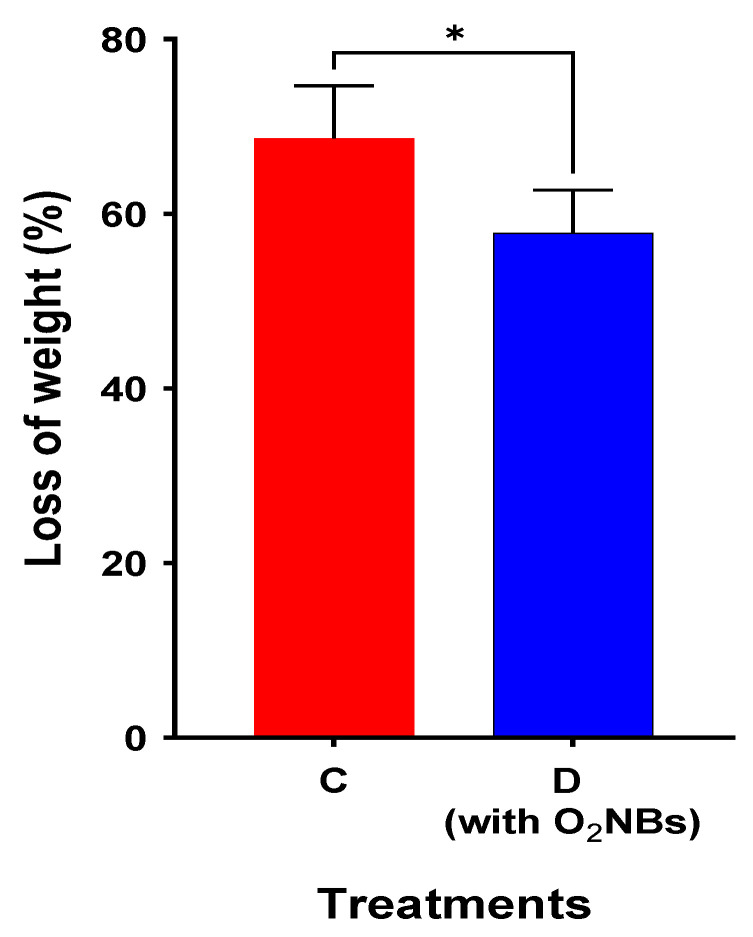
Comparison of loss of weight for the treatments irrigated with and without O_2_NBs (star indicates the level of significance: * for *p* < 0.05).

Even though the results demonstrate that there is no increase of bioaccumulation with the addition of OAs at a low concentration, it is worth investigating whether the nanobubbles have a significant impact on the phytochemical responses of the plant and the metal uptake. A t-test between the two treatments (C and D) was performed and a statistically significant difference between the two treatments was confirmed (level of significance 0.05), as shown in Figure 7.

**Figure 8:**
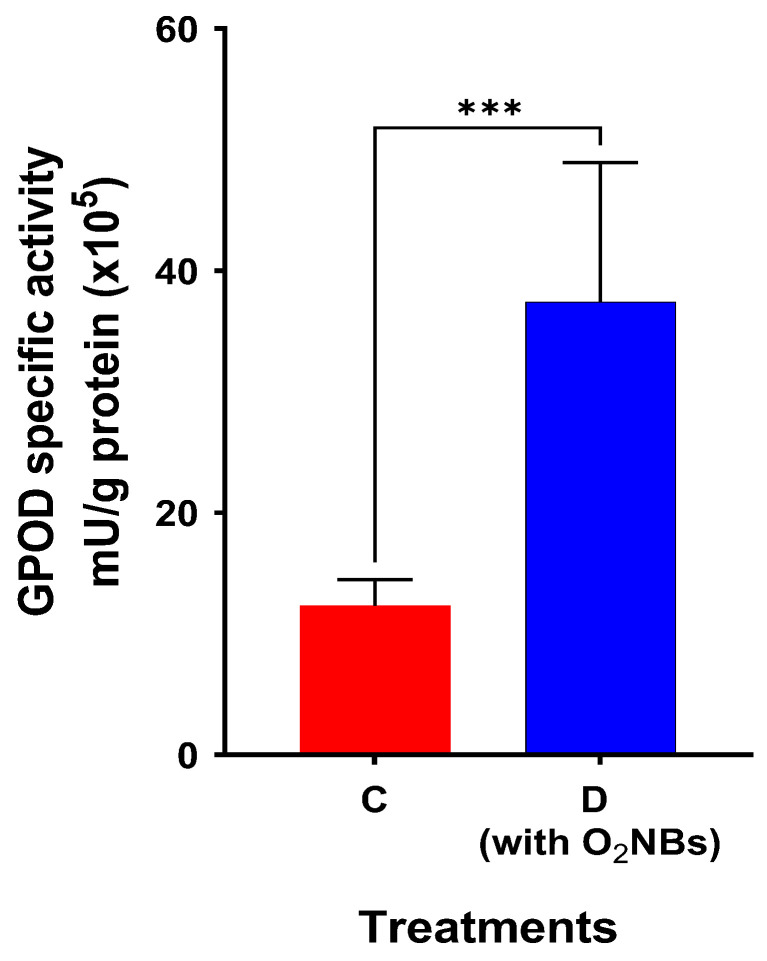
Comparison of GPOD activity in roots per g-protein in the treatments with and without O_2_NBs (star indicates the level of significance: *** for *p* < 0.001).

Treatment D, which contains O_2_NBs, exhibited a loss of weight that is statistically lower than those in all other treatments without the presence of nanobubbles. Moreover, as shown in Figure 8, a statistically significant increase of GPOD activity in the roots was observed between treatments C and D. In the presence of O_2_NBs, the antioxidant activity was statistically significantly higher.

**Figure 9:**
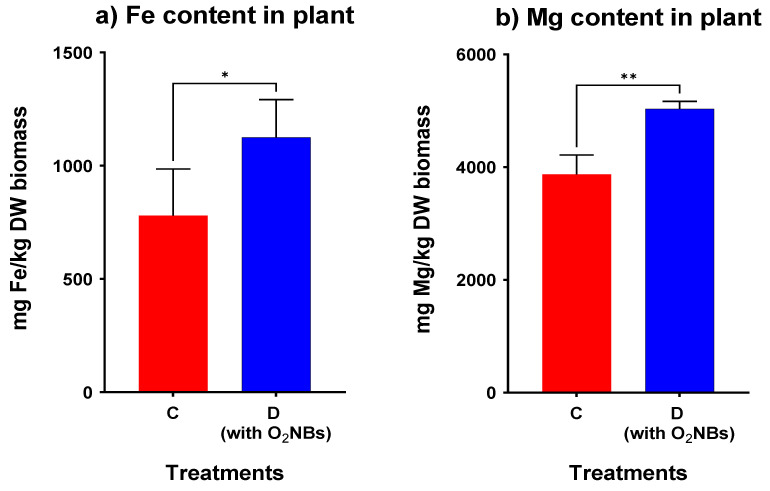

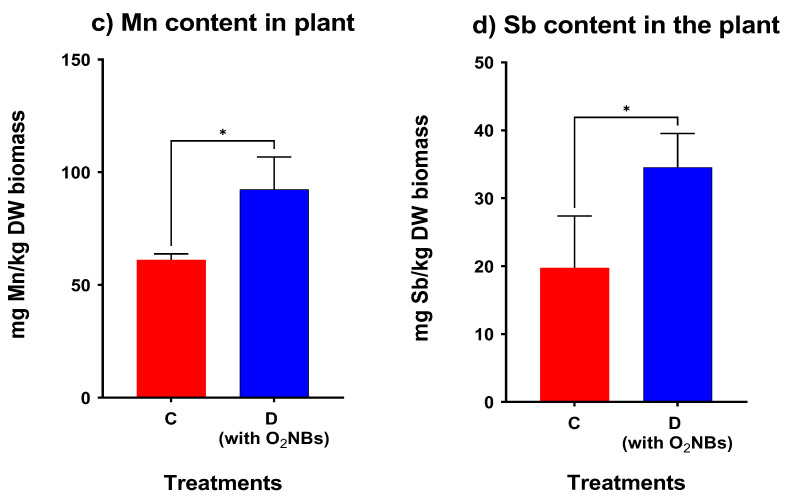
Comparison of total accumulation of (**a**) Fe, (**b**) Mg, (**c**) Mn and (**d**) Sb in plant tissues for treatments with and without O_2_NBs (Star indicates the level of significance: * for *p* < 0.05, ** for *p* < 0.01).

As seen in Figure 9, in treatment D containing nanobubbles, the accumulation of Fe, Mn, Mg, and Sb from the soil to the plant was enhanced, as each metal concentration (mg/kg DW biomass) was found to be significantly higher in treatment D, which was irrigated with nanobubbles.

## 3. Discussion

### 3.1. Sb Uptake by N. oleander

Plant growth was monitored in soil collected from the Kampani (Chania, Greece) shooting range (background level 1.17 ppm; treatment A) and the same soil was spiked with Sb(III) to bring the soil concentration to 50 ppm (treatments B, C, D & E). We have chosen to utilize this substrate, even though it had a low Sb concentration, in order to maintain the soil properties and all other existing elements in the soil (Fe, Mg, Mn, Co, Cu, Zn, Pb, and As). Soil samples were spiked with Sb (final concentration 50 ppm) and the experiments were conducted after a short period of time allowed for weathering. The bioavailability of Sb is relatively high since no long-term chemical processes occurred to decrease the bioavailable Sb content. Given the increased bioavailability of Sb in our experiments, the investigation of Sb uptake by *N. oleander* represents the maximum compared to long-term weathered soils with similar Sb content. This information is useful, given that no information on the phytoremediation capacity of *N. oleander* in Sb-contaminated soil is available in the literature.

As shown in Figure 2, plant growth of *N. oleander* was inhibited when cultivated in soil collected from the shooting range and spiked with Sb. The highest percentage loss was recorded for treatment C. A decrease of weight has also been observed for maize, as reported by Pan et al. [39]. On the contrary, in our study, the chlorophyll content (Figure 3) was not significantly affected by the concentration of 50 mg-Sb/kg, whereas there was a significant increase of GPOD activity in the roots for treatments D and E, where the Sb accumulation was the highest among the treatments. An increase of the antioxidant response of leaves was found in treatments C and D. The catalase activity was practically the same (Figure 4) for all treatments.

Soil pH is a crucial factor for Sb adsorption since it can influence its mobility [40]. OAs can acidify rhizosphere’s pH and chelate metals by forming complexes. In an acidic soil, leaching can be increased due to the high mobility of organic matter, leading to metal release [41]. Organic anions may also increase the pH because of the mineralization of organic anion to CO_2_ and water or the alkaline nature of the organic material. Furthermore, microbial decarboxylation of OA anions can result in a pH increase due to H^+^ consumption, as well as due to the formation of CO_2_ and O_2_ [42]. This is consistent with the measurements of pH in this work (Table 1), since all treatments showed an increase of pH, while a decrease of pH was observed only in the control.

### 3.2. Effect of Supplementation with Organic Acids

The addition of OAs in low concentrations had no significant effect on metal uptake, since the bioaccumulation factor was estimated below 1 and no translocation to the aboveground tissues was recorded. As reported in the literature, heavy metals solubility in soil is not significantly affected by the addition of OAs [43]. The lowest Sb concentration in plants was noticed in treatment C, where the low concentration of OAs was used. This can be elucidated by the fact that the Fe content was significantly low, according to Figure 6a. In general, the iron compounds are the predominant binders for trace elements in soils. Precisely, the iron oxides play a key role since they exhibit a binding activity to heavy metals due to their adsorption capacity. The corresponding low accumulation of Sb in OAs treatment appears to suggest a link between low iron solubility and low mobility of Sb(III) in this soil. This issue has been considered by Onireti et al., who highlight that even though oxalic acid can contribute in the dissolution of iron, when its concentration is insufficient, insoluble iron oxalates are formed [44]. Therefore, it can be inferred that the concentration of 7 mmol/ kg was too low to mobilize Fe and hence, also Sb.

On the other hand, when a high concentration of OAs was added (treatment E), a BCF above 1.0 was measured, indicating higher bioaccumulation in roots. Additionally, in this case, no significant translocation occurred from roots to stems and leaves. Examining the Fe content in plants, it can be seen that in this treatment, the highest Fe accumulation was reported among the treatments. Thus, the link between the Fe and Sb content can be confirmed anew, suggesting the utilization of a high concentration of OAs to ascertain Sb extraction from soil to plant.

### 3.3. Effect of Supplementation with Oxygen Nanobubbles

The combined use of OAs and O_2_NBs assisted the plant to maintain the water content in similar level to the control.

The capacity of the OAs to assist the plant to accumulate heavy metals increased significantly in the presence of O_2_NBs in the irrigation water. In the treatment where nanobubbles were used, the loss of weight was significantly lower. In addition, the application of O_2_NBs induced a significant increase of the GPOD activity of the root tissues. Regarding the metal concentration in the plant, it was found to be significantly greater for Sb but also for the metals Fe, Mn and Mg. However, in no case there was a high Sb extraction from the soil to the plant.

### 3.4. Phytoremediation Capacity of N. oleander

A previous study has reported a high translocation factor of Sb(III), indicating that Sb could be easily translocated from root to shoots in maize (*Zea mays*) and the accumulation of Sb was increased when the Sb concentration in soil was increased. However, the metal content in the roots was low compared to the initial soil concentration [39]. Similarly, a higher bioaccumulation of Sb in *Acorus calamus* was observed at higher Sb concentrations in soil, with the metal being accumulated primarily in the underground part of the plant [45]. In the present study, Sb was found mainly in the roots compared to the aboveground part. Moreover, the concentration of other metals within the *N. oleander* plants was found to be higher for the same initial soil Sb concentration compared to previous studies with other plants. Pan et al. [39] also used contaminated soils with 50 ppm Sb(III) and found that the overall accumulation of Sb in plant tissues was 8.82 mg/kg DW biomass of maize plant, as compared to 19.73 mg/kg DW biomass that we observed in our experiment as the lowest Sb content for *N. oleander* (treatment B). Furthermore, in soil with five times more Sb(III), *A. calamus* accumulated 39.72 mg/kg DW biomass in the underground part, which is lower than the highest Sb content (61.48 mg/kg DW) obtained in our study. Even though the bioconcentration is below the value of 1.0, Sb concentration in *N. oleander* was found to be higher compared to the reported concentrations of Sb in other plants, as discussed above.

It is well known that *N. oleander* is an ornamental plant that can be adapted to extreme drought and other stressors, and is widely selected in phytoremediation applications, since it reduces HMs from various matrices through phytoaccumulation and phytostabilization. This has been confirmed by several studies in the literature, indicating that *N. oleander* is a suitable plant species for biomonitoring of heavy metal pollution [46,47,48,49,50]. It is clear that each metal has a different uptake mechanism, which is also influenced by the soil characteristics. For instance, Pb was accumulated in the root, while Cd and Zn were readily translocated to the aerial compartment of the *N. oleander* plant [24]. Previous research exhibited TF > 1 for Cd and Pb in two different concentrations, indicating that translocation of metals was achieved, while no significant accumulation from soil to the roots and shoots of the plant was observed [51]. BCFs were evaluated as low for Al, Cd, Cu, Fe, and Pb, however, TF > 1 for these elements were estimated in mining and control areas. [52]. On the contrary, another study reports that the trace elements Cd, Zn, and Pb were highly concentrated in the root system compared to the aerial part [53]. These findings were confirmed by Elloumi et al., who indicated that the HMs (Zn, Ni and Cr) were immobilized in the roots and displayed a very low translocation factor [54]. Although research has illuminated the phytoremediation potential of *N. oleander* for various HMs, no study to date has examined Sb uptake. Therefore, it is important to note any study focusing on arsenic (As) phytoremediation, since As belongs to the same group of the periodic table (15) as antimony and they share similar chemical properties. It was reported that the translocation factor and the bioaccumulation values of As in *N. oleander* are 0.51 ± 0.25 and 0.13 ± 0.01, respectively, at an initial arsenic dose of 100 μM [49]. In our experiments, the Sb concentration in the roots was higher than in the above ground part for all treatments. Furthermore, Sb could not be translocated from roots to stems and leaves, contrary to the behaviour of other HMs, which exhibited considerable translocation, as previously mentioned. The highest TF value (0.23) was found in treatment C, whilst in all other treatments, TF was very low (<0.1). Regarding the other elements investigated in this study (Fe, Mg, and Mn), the results are similar, as the concentration is substantially higher in the roots compared to the aboveground tissue, except for the Mn content in treatment C. In this instance, the TF was estimated to be greater than 1. With respect to the Sb content in roots, this differed among the treatments. Even though the value of BCF was estimated to be less than one in most treatments, in treatment E (with the highest concentration of OAs), the Sb content in plant was higher than those in soil, rendering *N. oleander* an accumulator of Sb. Finally, it should be emphasized that our findings represent the maximum phytoremediation capacity of *N. oleander* for Sb since the Sb was spiked and not weathered for a long time.

## 4. Materials and Methods

### 4.1. Soil Characterization

Soil was collected from a shooting range in the Kampani area (Chania, Greece). The soil was passed through a 2 mm-sieve and was analyzed in terms of metals content. Since the antimony level was found to be very low (~1.17 ppm), laboratory spiking with Sb was performed [55]. In particular, potassium antimonyl tartrate trihydrate (C_8_H_4_K_2_O_12_Sb_2_·3 H_2_O) was added to achieve the desired initial antimony concentration of 50 ppm. Soil pH was measured using 10 g air-dried soil, adding 25 mL 1 M KCl [56]. Particle size was measured by the soil hydrometer, Bouyoucos [57]. The physical and chemical properties of the spiked soil are listed in Table 3.

### 4.2. Soil Amendements

#### 4.2.1. Organic Acids

The addition of organic acids aimed to decrease the pH below the initial value of 7.42. Due to the strong buffer capacity of soil, the concentration that achieved this decrease was 7 mmol/kg, while ten times higher concentration was also tested. A solution of citric acid (CA), oxalic acid (OA), and ascorbic acid (AA) was added to pots four times in low concentration of 7 mmol/kg (mass of acid /mass of soil) and two times in high concentration of 70 mmol/kg during the period of the experiment.

#### 4.2.2. Oxygen Nanobubbles (O_2_NBs) Production

O_2_NBs were prepared by the commercially available MK1 Nanobubbler^TM^ (Fine Bubble Technologies Pty Ltd., Cape Town, South Africa). The device was submerged in a 350 L water tank and was operated for 20 min with high-purity oxygen (99.9%) as feed gas before each irrigation event. In order to ensure that irrigation water did not contain any large bubbles, the NBs water was collected 10 min after the NB generation was completed, in order to allow any larger bubbles to come up to the surface, where they burst out. Samples from the tank were used to obtain the NBs density using nanoparticle tracking analysis (NTA) (Nanosight, Malvern, UK) and the average diameter size combining dynamic light scattering (DLS) (Sald 7500 nano, Shimadzu, Kyoto, Japan) and NTA analysis. The average particle size and the concentration were found to be 175 ± 17 nm and 2.1 × 10^7^ ± 6.8 × 10^6^ particles/mL, respectively. The estimated oxygen concentration was found to be five times higher than oxygen solubility in equilibrium.

### 4.3. Pot Experiment

The phytoremediation potential of *Nerium oleander* was investigated by carrying out a pot experiment for a period of 6 weeks in the greenhouse at the Technical University of Crete (Chania, Greece) under ambient air with protection against rain. Six-month-old plants were picked from a nursery garden in the Kounoupidiana district, Chania, and were divided into 5 experimental groups with similar total biomass (weight and height) in order to assure homogeneity among the treatments. The detailed experimental design is shown in Table 4. Specifically, each treatment had four replicates, resulting in a total of 20 pots being used. Plants were watered every 2–3 days, depending on the soil moisture content, with approximately 50–100 L of tap water. In the case of the treatment D, plants were irrigated with tap water containing O_2_NBs. Finally, plastic trays were placed under the pots to avoid any water leakage, and hence, any metal loss.

### 4.4. Chlorophyll Measurements

At the end of the experiment, representative samples of each plant condition were collected to estimate the chlorophyll content [58]. Leaf samples (0.2 g) were collected and grounded in a ceramic mortar with 10 mL of 80% acetone. The absorbance of the supernatant after centrifugation was measured at 663 and 646 nm using a UV–VIS spectrometer to determine chlorophyll a, chlorophyll b, and total chlorophyll concentrations.

### 4.5. Measurement of Antioxidant Enzymes Activity

For extraction of enzymes, 1 g of fresh leaves and roots was ground and homogenized in a potassium phosphate buffer 100 mM (pH = 7) containing 0.1 mM EDTA and 1% (w/v) PVP. The extract was filtered through multiple layers of cheesecloth and the supernatant was centrifuged at 16,000× *g* for 25 min. Protein concentration was determined using the Bradford assay [59]. The activity of guaiacol-peroxidase (GPOD) was determined by monitoring the increase of absorbance due to the oxidation of guaiacol at 470 nm for 3 min using a UV–vis spectrometer (coefficient of absorbance, ε = 25.5 mM^−1^cm^−1^). Briefly, a reaction mixture was prepared by phosphate buffer (50 mM, pH = 5.8), guaiacol (15 mg/mL), a suitable amount of plant extract and H_2_O_2_ (1% v/v) [60]. The activity of catalase (CAT) was determined recording the decrease of absorbance as a result of H_2_O_2_ degradation at 240 nm for 3 min (coefficient of absorbance, ε = 43.6 mM^−1^cm^−1^). The extraction mixture contained phosphate buffer (50 mM, pH = 7), H_2_O_2_ (36 mM) and a suitable aliquot of supernatant enzyme [61]. The enzymes activity unit was expressed as the change in absorbance per minute in terms of units per milligram of extracted proteins.

### 4.6. Water Content and Biomass Measurement

At the beginning of the experiment, the fresh weight of the plants was measured. After the experimental period, entire plants were carefully taken out of the soil and washed with tap water, then rinsed twice with deionized water to remove any dust/dirt. Then, they were separated into roots and shoots, and their fresh weights (FW) were determined. Dry weights (DW) were determined after oven drying for 48 h at 70 °C and cooled down to room temperature. Water content (WC, %) was estimated according to the equation below:

$$\mathrm{WC}\left(\%\right)=\frac{\mathrm{FW}-\mathrm{DW}}{\mathrm{FW}}\times 100\%$$

The loss total weight was estimated from the weight of the plants before (FW_o_) and after (FW_t_) the experiment, based on the following formula:

$$\mathrm{Loss}\;\mathrm{of}\;\mathrm{total}\;\mathrm{weight}=\frac{{\mathrm{FW}}_{o}-{\mathrm{FW}}_{t}}{{\mathrm{FW}}_{o}}$$

### 4.7. Heavy Metal Analysis

Plants tissues were air dried (48 h at 70 °C) and digested for the metal content determination. 0.5 g of milled plant samples were ashed in the muffle furnace for 16 h at 480 °C, then dissolved with 1.5 mL citric acid (5 M) and 7.5 mL HNO_3_ (>69%) on a hot plate (~100 °C). The solution was diluted with ultrapure water to 45 mL and agitated for 24 h. Afterwards, the samples were filtered (0.45 μm, Whatman) and analyzed by ICP-MS. In parallel, the soil metal content determination was performed. The total amount of soil was collected from the pots, air dried in plastic bags, and passed once more through a 2 mm mesh size sieve. Soil samples (0.2 g) were treated with citric acid (5 M) and HNO_3_ (>69%) following the aforementioned procedure.

### 4.8. Bioaccumulation Factor (BC) and Translocation Factor (TF)

The evaluation of the metal accumulation efficiency of the *N. oleander* was assessed by estimating two main parameters: the bioconcentration factor and translocation factor, according to the following equations.

$$\mathrm{BCF}=\frac{C_{\mathrm{Plant}}}{C_{\mathrm{Soil}}}$$

where C_Plant_ is the metal concentration in the plant (roots and shoots) and C_Soil_ is the metal concentration in the soil after the culture experiment.

$$\mathrm{TF}=\frac{C_{\mathrm{Shoots}}}{C_{\mathrm{Root}}}$$

where C_Shoots_ is the metal concentration in the shoots and C_Root_ is the metal concentration in the roots after the culture experiment. BCF is expressed as the ratio of metal in the plant to that in soil, while TF is expressed as the ratio of the metal in the aerial parts to the roots. A BCF value higher than one indicates that a plant is an accumulator, and a TF value higher than one is indicative of a high translocation ability of metals from roots to shoots.

### 4.9. Quality Control and Statistical Analysis

Triplicate measurements in the extracts, measurement of calibration blanks, laboratory reagent blanks, as well as analysis of standard reference material were employed in order to address data quality control. All data are presented as mean ± standard deviation. Statistical analysis was performed using GraphPad Prism 9 software. Data variation was analyzed with one-way analysis of variance (ANOVA) at significant level of *p* < 0.05.

## 5. Conclusions

Our experimental results with *N. oleander* revealed that the use of organic acids (OAs) can have a significant effect on Sb accumulation when an appropriate concentration of OAs is applied. Specifically, the low concentration of OAs (7 mmol/kg) did not show a significant effect on Sb accumulation compared to the control. The bioconcentration and translocation factors were estimated to be below one, revealing that there was no high extraction from the soil to the plant or translocation from the roots to the leaves of *N. oleander*, respectively. On the contrary, in the treatment with the highest concentration of OAs (70 mmol/kg), the bioconcentration factor was estimated to be above one, leading to the conclusion that the bioaccumulation of Sb from soil to the plant tissues was significantly enhanced. In addition, it was demonstrated that oxygen nanobubbles (O_2_NBs) can increase the Sb uptake in addition to the increased accumulation of other metals (Fe, Mg, and Mn).

Overall, the use of *N. oleander* combined with supplementation with organic acids at a high concentration resulted in a high bioaccumulation of Sb, preferentially in the roots, making this plant suitable for the phytostabilization of Sb-contaminated soils.

## Figures and Tables

**Table 1:** Measurement of pH before and after the experiment for all treatments.

pH			Treatment		
	A (Control)	B Sb 50 ppm	C Sb 50 ppm OAs 7 mmol/kg	D Sb 50ppm OAs 7 mmol/kg with O_2_NBs	E Sb 50 ppm OAs 70 mmol/kg
Before	7.42	7.42	6.61	6.61	6.04
After	7.17	7.51	7.65	7.61	7.23

**Table 2:** Sb bioconcentration factor (BCF) and translocation factor (TF) for all treatments (star indicates the level of significance: * for *p* < 0.05, **** for *p* < 0.0001).

Factors		Treatment		
	B	C	D	E
BCF	0.78	0.51	0.90	1.72 *
TF	0.07	0.23 ****	0.09	0.06

**Table 3:** Physical and chemical characteristics of the soil used in this study.

Soil Property	Value
Sand (%)	72.53
Clay (%)	21.87
Slit (%)	5.6
Texture	sand clay loam
Organic matter (%)	1.83
TKN (g/kg soil)	0.76
pH	7.42
Sb concentration (ppm)	48.8 ± 1.3

**Table 4:** Experimental Design.

Experimental Treatment (Code Name)	Sb Concentration [ppm]	Organic Acids Concentration [mmol/kg]	O_2_NBs
A (control)	1.17 ppm (background level)	0	-
B	50	0	-
C	50	7	-
D	50	7	+
E	50	70	-

## Data Availability

Data available upon request.
